# Dietary fat and not calcium supplementation or dairy product consumption is associated with changes in anthropometrics during a randomized, placebo-controlled energy-restriction trial

**DOI:** 10.1186/1743-7075-8-67

**Published:** 2011-10-05

**Authors:** Jennifer T Smilowitz, Michelle M Wiest, Dorothy Teegarden, Michael B Zemel, J Bruce German, Marta D Van Loan

**Affiliations:** 1 Department of Food Science and Technology, University of California, Davis, CA 95616, USA; 2 Foods for Health Institute, University of California Davis, Davis, CA 95616, USA; 3 Department of Statistics, University of Idaho, Moscow, ID 83844, USA; 4 Department of Food and Nutrition, Purdue University, W. Lafayette, IN 47907, USA; 5 Department of Nutrition, University of Tennessee, Knoxville, TN 37996, USA; 6 USDA, ARS, Western Human Nutrition Research Center, Davis, CA 95616, USA

## Abstract

Insufficient calcium intake has been proposed to cause unbalanced energy partitioning leading to obesity. However, weight loss interventions including dietary calcium or dairy product consumption have not reported changes in lipid metabolism measured by the plasma lipidome.

**Methods:**

The objective of this study was to determine the relationships between dairy product or supplemental calcium intake with changes in the plasma lipidome and body composition during energy restriction. A secondary objective of this study was to explore the relationships among calculated macronutrient composition of the energy restricted diet to changes in the plasma lipidome, and body composition during energy restriction. Overweight adults (n = 61) were randomized into one of three intervention groups including a deficit of 500kcal/d: 1) placebo; 2) 900 mg/d calcium supplement; and 3) 3-4 servings of dairy products/d plus a placebo supplement. Plasma fatty acid methyl esters of cholesterol ester, diacylglycerol, free fatty acids, lysophosphatidylcholine, phosphatidylcholine, phosphatidylethanolamine and triacylglycerol were quantified by capillary gas chromatography.

**Results:**

After adjustments for energy and protein (g/d) intake, there was no significant effect of treatment on changes in weight, waist circumference or body composition. Plasma lipidome did not differ among dietary treatment groups. Stepwise regression identified correlations between reported intake of monounsaturated fat (% of energy) and changes in % lean mass (r = -0.44, *P *< 0.01) and % body fat (r = 0.48, *P *< 0.001). Polyunsaturated fat intake was associated with the % change in waist circumference (r = 0.44, P < 0.01). Dietary saturated fat was not associated with any changes in anthropometrics or the plasma lipidome.

**Conclusions:**

Dairy product consumption or calcium supplementation during energy restriction over the course of 12 weeks did not affect plasma lipids. Independent of calcium and dairy product consumption, short-term energy restriction altered body composition. Reported dietary fat composition of energy restricted diets was associated with the degree of change in body composition in these overweight and obese individuals.

## Background

Chronic unfavorable alterations in metabolic regulation eventually lead to life-threatening diseases characterized symptomatically by abdominal obesity, dyslipidemia, hypertension, insulin resistance, type 2 diabetes, and coronary artery disease [1]. Metabolic dysregulation and its consequences have been assigned to genetic predispositions combined with environmental and dietary factors that in some individuals lead to unbalanced energy intake and expenditure. Diet, both essential and nonessential nutrients may play a role in metabolic regulation [2]. Particular studies suggest that insufficient calcium intake is associated with unbalanced lipid metabolism in children and adults [3-5]. Mean dietary intakes of calcium in the US adult population are below the adequate intake values [6] and milk intake has also decreased over the past three decades [7]. Observational and randomized clinical studies demonstrate significant inverse associations between calcium [8-12] and dairy product intake [7,8,10,13], and both body fat (BF) mass and body weight. During energy restriction, calcium supplementation augmented weight and BF losses compared with the control group without supplementation [4,5]. Yet, dairy product consumption accelerated weight and BF losses to a greater degree than supplemental calcium [3]. How dairy composition and structure modulates lipid metabolism is not well understood.

Lipid metabolism can be estimated by accurate measures of tissue lipid metabolites in which concentrations of substrates and products reflect entire biochemical pathways [14]. Tissue and circulating lipidome reflect genetics [15], diet [16-20], lipid metabolism [21-25], stress [26] and endocrine activity [24]. Several plasma lipid metabolites are maintained normally within narrow concentrations. Measured deviations from normal ranges are being recognized as diagnostic indices for both early and late stages of metabolic dysregulation. For example, elevated concentrations of serum total free fatty acids (FFA), and of palmitic acid (16:0) in particular are associated with insulin resistance in peripheral tissues including muscle, liver, and pancreas [27]. Moreover, independent prospective case-cohort studies have found that the incidence of type 2 diabetes was positively associated with the proportions of plasma palmitic (16:0) and stearic acid (18:0) in middle aged men and women [28,29]. In overweight otherwise healthy individuals, baseline circulating phosphatidylcholine (PC) stearic acid (18:0), and FFA 18:1(n-9) explained 20-30% of the variance for changes in body composition during energy restriction in those individuals that lost weight [25]. Use of quantitative lipidomics led to the identification of plasma palmitoleate (16:1n7) as an adipose tissue-derived lipid hormone that strongly stimulated muscle insulin action and suppressed hepatosteatosis in adipose tissue lipid chaperone aP2 and mal1 deficient mice [24]. The primary aim of this of this study was to determine the relationship between dairy product or supplemental calcium intake and changes in the plasma lipidome and body composition of overweight and obese individuals during energy restriction. An additional aim examined associations between reported compositions of energy restricted diets independent of treatment group, plasma lipidome measurements and changes in anthropometrics.

## Methods

### Subjects

In the parent project 105 overweight and mildly obese individuals were recruited from the faculty, staff, and student populations of each of the three participating institutions (University of Tennessee, Purdue University, and the USDA, ARS, Western Human Nutrition Research Center at the University of California, Davis). Seventy nine subjects completed the trial and 63 met the parent study's a priori weekly compliance criteria [30], however due to missing measurements, the majority of data are reported for 61 subjects. Intent-to-treat analysis was not conducted because there were only two time points: baseline and 12 weeks. Subjects were included in the study if they were: 18-35 years of age, had an initial BMI of 25-34.9 kg/m^2^, consumed a low-calcium diet at enrollment (< 600 mg calcium/d) from non-calcium-fortified foods and < 800 mg total calcium/d, did not gain or lose more than 3 kg of body weight during the past three months, and did not recently (4 wk) change exercise intensity or frequency. Subjects were excluded from participation of the study if they smoked; required the use of oral anti-diabetic agents or insulin; used obesity pharmacotherapeutic agents and/or herbal or other preparations intended for use in obesity or weight management within the previous 12 wk; used calcium supplements within the previous 12 wk; had a history of significant endocrine, hepatic, or renal disease; were pregnant or lactating; had a recent (past 12 wk) initiation of or change in oral contraceptive; suffered any active form of malabsorption syndrome; or had a history of eating disorders. The project was approved by the Institutional Review Board of each of the three participating institutions. Written informed consent was obtained from all subjects, and the research was conducted in accordance with the ethical standards outlined in the Helsinki Declaration.

### Study Protocol

This study was designed to determine whether dairy products or calcium supplementation would accelerate weight and fat loss induced by energy restriction in otherwise healthy overweight and obese adults. After enrollment, subjects were studied for a 2-wk lead-in period to establish their current energy requirements and provide an opportunity for baseline dietary and physiological assessment, and then randomized to the following dietary regimens for 12 wk: 1) a control diet providing a 2093 kJ/d deficit (500 kcal/d), 0-1 serving of dairy products/d, 500 mg calcium/d, and a daily placebo supplement; 2) a calcium-supplemented diet identical to the control diet, with the placebo replaced by 900 mg calcium in the form of calcium carbonate; or 3) a high-dairy diet (placebo-supplemented) providing a 2093 kJ/d deficit (500 kcal/d) and containing three daily servings dairy products (milk, cheese, and/or yogurt) to bring the total calcium intake from 500 to 1400 mg/d. The calcium supplemented and placebo arms of the study were conducted in a placebo-controlled, blinded fashion, and the dairy product arm was unblinded by necessity. However, subjects on the high-dairy diet also received a placebo supplement and all groups were treated as active-treatment arms, with pill counts serving as a compliance measurement.

### Diets

Following instructions from a trained nutritionist during the 2-wk lead-in period, baseline 7-d dietary records were completed by each subject and reviewed for completeness by the project dietitian at each site. Diet records were analyzed by Nutritionist Pro software and were used to provide an initial estimate of an energy intake. The estimate of energy intake was then refined by calculating energy needs using World Health Organization equations for calculating basal metabolic rate, adjusted for Physical Activity Level (PAL) set at low-active (1.4) to provide an estimate of total daily energy expenditure, and reported elsewhere [31]. Based on this initial estimate of energy needs, a food exchange-based diet was prescribed to produce an energy deficit of approximately 2093 kJ/d. The diets for the treatment arms were constructed to provide comparable levels of macronutrient and fiber, to approximate the average consumption in the U.S. (fat, ~35% of total energy, carbohydrates ~49%, ~protein 16%, fiber 2-3 g/1,000 kJ/day). Nutritional supplements were not permitted, and caffeine intake was maintained at a constant level (individualized for each patient, based on baseline assessment). Diets were prescribed and monitored as noted above. Subjects in the high dairy group were permitted to utilize both full-fat and low-fat milk, cheese and yogurt, with the fat accounted for in exchange lists given with each individual diet prescription. Subjects were provided individual instruction, counseling, and assessment from the study dietitian regarding dietary adherence and the development and reinforcement of strategies for continued success. Subjects maintained daily food records throughout the 12 wk intervention period, and compliance was assessed by weekly subject interview and review of the diet diary and pill counts. Compliance criteria are reported elsewhere [30,31].

Nutrient assessment was computed by averaging 7-d diet records for the 2-wk run-in period with each day of the week represented twice. During the intervention weeks 0-11, subjects filled out 7-day diet records but nutrient intake was assessed from averaging data from 3 of the 7 days (2 progressive weekdays and 1 weekend). The 3 day selection from each week (0-11) started with the first weekday and alternated between Saturday and Sunday. Each 3 days rotated through the week such that by the end of the 12wk intervention, a total of 36 days were included with each day of the week counted 3 times. For example, during week 0, nutrient intake data were selected from Monday, Tuesday, and Saturday and during week 1, nutrient intake data were selected for Wednesday, Thursday and Sunday. Nutrient analysis was completed using Nutritionist Pro Food Processor Plus software. Baseline micro- and macronutrient intakes were determined by averaging the pooled nutrient data from the 2-wk run-in period. Micro- and macronutrient intake during the 12 wk intervention period was determined by averaging the pooled nutrient data from weeks 2-11. Data were not included for baseline or week 1 to allow subjects a 2-wk adaptation period to accurately log dietary intake data.

### Anthropometric measurements

Measurement of body weight was done during the 2-wk run-in period and weekly thereafter; height was measured at baseline, and waist circumference (WC) at baseline and 12 wk. BMI was calculated as kg/m^2^. Total fat and lean mass (LM) were assessed via dual energy X-ray absorptiometry (Lunar Prodigy instrument; GE Medical Instruments) at baseline and week 12 of the study. Statistical models were developed to predict the following anthropometric outcomes: 1) percent change in weight; 2) percent change in WC; 3) percent change in % LM and 4) percent change in % body fat (BF).

### Physical activity assessment

Participants were instructed not to change their usual physical activity habits during the study and to ensure that physical activity remained unchanged; 3-d physical activity records were collected from all subjects at baseline and week 12. Participants were asked to report any changes to their usual physical activity.

### Metabolite measurements

Plasma glucose, TG, LDL cholesterol, and HDL cholesterol were measured at each site's clinical medical laboratory with a Beckman Lxi-725 auto-analyzer. Fasting plasma insulin was measured using a commercially available radioimmunoassay kit (Linco Research, St. Charles, MO) at each site's clinical medical laboratory. Insulin resistance at baseline and 12 wk was calculated using the Homeostasis Model Assessment (HOMA-IR) [32].

### Analysis of the plasma lipidome

Blood was collected from all participants into evacuated tubes containing EDTA, centrifuged immediately (1300 × g, 10 min, 20°C), portioned into aliquots, and stored at -80°C until analyzed. Fatty acid analyses of circulating lipid classes were measured by high-throughput methods developed by Lipomics Technologies, Inc. (West Sacramento, CA) [33]. In brief, the lipids from plasma (200 μl) were extracted using a modified Folch extraction in chloroform:methanol (2:1 v/v) [34]. Each extraction was performed in the presence of a panel of quantitative authentic internal standards. Extracted lipids were dried under N_2 _gas for for separation by chromatography. Individual phospholipid classes within the extract were separated by high performance liquid chromatography [24]. Individual neutral lipid classes were separated from the extract by thin layer chromatography. Each separated lipid class was collected and dried under nitrogen in preparation for trans-esterification. Lipid classes were trans-esterified in 3 N methanolic HCl in sealed vials under a nitrogen atmosphere at 100°C for 45 min. The resulting fatty acid methyl esters were extracted from the mixture with hexane and prepared for automatic injection into a gas chromatograph by sealing the hexane extracts under nitrogen. Fatty acid methyl esters were separated and quantified by capillary GC using an Agilent 6890 gas chromatograph equipped with a 30-m DB-88 capillary column (Agilent Technologies, Santa Clara, CA) and a flame-ionization detector. Fatty acids of each lipid class were determined quantitatively (μmol/L) and expressed as a percentage of total fatty acids within that class (mol %). Total lipid classes were calculated as the sum of individual fatty acid species for each lipid class fraction. Fatty acids with missing values at 20% or greater were dropped from the analyses and considered not determined (ND).

### Statistical analyses

All statistical procedures were conducted using SPSS version 16 for Windows (SPSS, Chicago, IL). Means ± SD are reported for baseline and 12 wk anthropometric measurements; reported macro- and micronutrient intake; circulating plasma clinical metabolites; circulating total lipid classes; and fatty acid composition of circulating cholesterol ester (CE), free fatty acids (FFA), phosphatidylcholine (PC), and triacylglycerol (TG). All data were examined for normality and transformed as needed prior to conducting statistical analyses. Repeated Measures ANOVA was performed to determine the effect of time, treatment and time x treatment on anthropometric outcomes, dietary intake and circulating lipids. If repeated measures ANOVA demonstrated a significant time effect between baseline and 12 wk variables, a paired sample two-tailed t-test was performed to identify the treatment group that reached significance. ANCOVA was performed to determine differences between treatment groups for both baseline and 12 wk circulating lipid metabolites, clinical metabolites, dietary intake, and anthropometric measurements. Multiple comparisons analysis with a Bonferroni adjustment was used to determine differences among the three treatment groups. Final models on dietary intake were adjusted for site, sex, and energy intake at 12 wk. Models for dependent variables that already included energy in their calculations--Protein, SFA, MUFA and PUFA (% of energy)--were not adjusted for 12wk energy intake. Stepwise regressions were generated to explore the relationships between 12 wk dietary fat composition and 12 wk circulating lipids of the same family (i.e., dietary saturated fat against circulating saturated fatty acids). Details of the criteria followed to generate stepwise regressions are reported elsewhere [25]. Stepwise regressions were also used to examine the relationships among 12 wk diet composition; plasma clinical biomarkers; and lipid metabolites with changes in anthropometrics. The three treatments and sites were converted into two orthogonal variables for use in linear regression. For each stepwise regression, the F statistic probability was set at an alpha between 0.01 and 0.05. Normality for each stepwise regression model was determined by generating normal probability plots of the regression standardized residual. Equal variance for each regression model across each dependent variable was determined by plotting the standardized predicted dependent variable by the standardized residuals. Outlying cases that strongly influenced each stepwise regression model were tested by Cook's distance (D). Data points with larger D values than the rest of the data were considered highly influential and deleted. The models with deleted observations with large D values were re-regressed and compared to ensure the model was statistically relevant and not a product of one highly influential data point. Multi-collinearity between baseline lipids selected by stepwise regression was checked by a Variance Inflation Factor of ≤ 2.0. If variables demonstrated a Variance Inflation Factor greater than 2.0, they were dropped from the final regression model.

The following stepwise regressions were generated: reported intake of % saturated fat (SFA) (% of fat) at 12 wk as a dependent variable and 12 wk circulating plasma 14:0, 15:0, 16:0, and 18:0 of each lipid class as independent variables; reported intake of % monounsaturated fat (MUFA) (% of fat) at 12 wk as a dependent variable and 12 wk circulating plasma 16:1(n-7), 18:1(n-7), 18:1(n-9) of each lipid class as independent variables; and reported intake of % polyunsaturated fat (PUFA) (% of fat) 12 wk as a dependent variable with 12 wk circulating plasma 18:2(n-6), 18:3(n-6), 20:3(n-6), 20:4(n-6), 18:3(n-3), 20:5(n-3), and 22:6(n-3) of each lipid class as independent variables. Stepwise regressions were also generated to determine the relationships between 12 wk dietary fat composition (SFA, MUFA, and PUFA) as a percent of energy and changes in body composition.

For each statistical analysis, only fatty acids for each lipid class with mean abundances of 1.0% or greater were analyzed. Estimation of desaturase and elongase enzymatic activities involved in fatty acid metabolism as product-to-precursor ratios were also analyzed. The ratios of circulating 16:1(n-7)/16:0 and 18:1(n-9)/18:0 were used as surrogates for delta 9-desaturase activity; the ratios of 18:3(n-6)/18:2(n-6) and 20:4(n-6)/20:3(n-6) were used as surrogates for delta 6- and delta 5-desaturase activity, respectively; and the ratios of 18:1(n-7)/16:1(n-7), 18:0/16:0, and 20:2(n-6)/18:2(n-6) were used as surrogates for elongase activities [35].

Fatty acid data were only available for 61 subjects; total lipid class data for 60 subjects; and plasma glucose, HOMA-IR and waist circumference at 12 wk were only available for 59 subjects and reported herein. ANCOVA and regressions used to determine relationships between any independent variable and change in anthropometrics were adjusted for site, age, sex, energy, protein (g/d) intakes and physical activity at 12 wk and baseline HOMA-IR.

## Results

### Anthropometric measurements at baseline and 12 wk for dairy, calcium, and placebo groups

Nineteen females and four males completed the dairy intervention, seventeen females and two males completed the calcium supplemental intervention, and twenty females and six males completed the placebo intervention (Table 1). There were no reported changes in physical activity during the study period and no significant differences for age, baseline weight, % LM, and % BF and WC among the treatment groups (data not shown). There were significant differences in the mean age (P < 0.0005), weight (P < 0.05), BMI (P < 0.05), waist circumference (P < 0.01), intakes of calcium (P < 0.05) and SFA (% of energy) (P < 0.01) at baseline and attrition rates (P < 0.0005) among the sites (data not shown). There were significant in reductions in weight, % BF, waist circumference, plasma insulin and HOMA-IR and an increase in % LM for all treatment groups at the end of the intervention (Table 1). The mean percent change in any anthropometric measurement was not significantly different among treatment groups after adjustments for dietary intake of protein, energy, and physical activity (data not shown).

**Table 1:** Anthropometric and clinical measurements of study participants at baseline and 12 wk for each treatment group^1^

	Dairy (n = 22 )		Calcium (n = 16)		Placebo (n = 23)	
**Characteristic**	**Baseline**	**12 wk**	**Baseline**	**12 wk**	**Baseline**	**12 wk**

Age (y)	25.1 ± 5.3		25.2 ± 4.9		24.2 ± 4.7	
Sex, No.(%)						
Female	18 (82)		14 (88)		18 (78)	
Male	4 (18)		2 (13)		5 (22)	
Height (cm)	166.9 ± 6.0		164.6 ± 9.0		166.1 ± 7.6	
Glucose (mmol/L) ^2,3^	4.6 ± 0.4	4.6 ± 0.4	4.8 ± 0.4	4.8 ± 0.5	4.8 ± 0.5	4.8 ± 0.4
Insulin (pmol/L) ^2^	42.5 ± 28.5	33.5 ± 29.0**	41.8 ± 26.2	35.1 ± 21.1**	35.7 ± 14.7	29.9 ± 10.3**
HOMA-IR^2,3^	1.3 ± 0.9	1.0 ± 0.9**	1.3 ± 0.9	1.1 ± 0.8**	1.1 ± 0.5	0.9 ± 0.3**
Weight (kg)^2^	79.1 ± 12.1	74.8 ± 10.4**	79.2 ± 12.6	75.8 ± 10.6**	79.7 ± 11.7	76.5 ± 11.2**
BMI^2^	28. 3 ± 3.0	26.7 ± 2.5**	29.1 ± 2.7	28.1 ± 2.6**	28.8 ± 2.7	27.7 ± 2.8**
LM (%)^2^	54.5 ± 5.9	57.9 ± 6.6**	58.3 ± 6.6	58.7 ± 7.0**	55.0 ± 5.9	56.9 ± 5.9**
BF (%)^2^	41.2 ± 5.7	37.7 ± 6.5**	37.6 ± 6.2	36.0 ± 6.7**	40.9 ± 5.2	38.8 ± 5.2**
WC (cm)^2,3^	88.9 ± 9.6	81.1 ± 8.6**	88.1 ± 9.2	83.3 ± 7.5**	87.4 ± 11.8	82.6 ± 11.3**

^1^Data are means ± SD; n = 61 unless otherwise indicated. WC, waist circumference; LM, lean mass. There were no significant differences in baseline anthropometrics or plasma metabolites among treatment groups (ANOVA).

^2^Repeated Measures ANOVA to test the effect of time; confirmed with paired sample t-test for significant differences between baseline and 12 wk, *P < 0.01, **P < 0.001, There was no significant effect of time or time x treatment interaction.

^3^n = 59.

### Mean dietary intake at baseline and 12 wk for dairy, calcium, and placebo groups

Total energy intake was significantly reduced from baseline for all treatment groups, yet the dairy group consumed about 15% more energy than the placebo and calcium groups (Table 2). In addition, the amount of protein (g/d) was reduced by 30% from baseline to 12 wk for the placebo and calcium groups, yet there was no difference for the dairy group (data not shown). Total dietary fat (% of energy) decreased from baseline to 12 wk for all of the treatment groups but the fat composition varied among the treatment groups. Intake of SFA (% of fat and energy) remained unchanged between baseline and 12wk for the dairy group but decreased for the calcium and placebo groups. The dairy group consumed approximately 25% higher saturated fat (% of fat) compared with the calcium and placebo groups. Intake of MUFA (% of fat) increased from baseline to 12 weeks for the calcium and placebo groups, but remained unchanged for the dairy group. The calcium and placebo groups consumed approximately 18% more monounsaturated fat (% of fat) compared with the dairy group. Intake of PUFA (% of fat and energy) remained unchanged between baseline and 12 wk among the treatment groups, however the calcium and placebo groups consumed approximately 24% more PUFA (% of fat) than the dairy group. The dairy group consumed fat with significantly lower n6:n3 compared with the calcium and placebo groups; however this relationship disappeared after adjustments for site, sex and energy. In addition to the macronutrients, there were significant differences for reported mean micronutrient intakes among the treatment groups. Reported dietary intakes of biotin, pantothenic acid, riboflavin, vitamin B12 and vitamin D were significantly higher in the dairy compared with the calcium and placebo groups. In addition, the dairy group reported greater intake of calcium by 20% and 1.8-fold compared with the calcium and placebo groups, respectively; and the calcium group reported a 1.3 fold higher intake of calcium compared with the placebo group. There were no significant differences among treatment groups for % carbohydrates (of energy), cholesterol, thiamin, and vitamin B_6 _(Table 2).

**Table 2:** Mean daily dietary intake at baseline and during the study (12 wk) for each treatment group^1^

	Dairy (n = 22)		Calcium (n = 16)		Placebo (n = 23)	
	
Nutrient	Baseline	12 wk	Baseline	12 wk	Baseline	12 wk
Total energy **(kcal/d) **^**2,4**^	2122 ± 630	1510 ± 225*^,a^	1910 ± 415	1300 ± 200*	1960 ± 500	1300 ± 200*
Energy **(kJ/d)**	8880 ± 2640	6340 ± 940	8000 ± 1740	5460 ± 850	8200 ± 2100	5440 ± 825
Carbohydrate **(% EN)**	51.8 ± 7.8	50.9 ± 5.4	48.7 ± 4.6	51.0 ± 4.8	52.6 ± 6.2	53.6 ± 5.2
Protein **(% EN) **^**2**^**^**^**, 4**^	14.0 ± 2.5	19.0 ± 1.8*^,a^	15.1 ± 2.5	17.4 ± 2.1*^, b^	14.4 ± 3.0	16.7 ± 2.5*^, b^
Fat **(% EN)**	31.8 ± 5.1	28.1 ± 4.7*	35.3 ± 4.9	30.1 ± 3.9*	31.9 ± 5.1	28.2 ± 4.7*
SFA **(% fat) **^**2**^**^**^**, 4**^	49.9 ± 5.8	50.5 ± 6.2^**a**^	44.9 ± 5.9	41.1 ± 3.6*^, b^	46.4 ± 5.0	41.3 ± 3.9*^, b^
SFA **(%EN) **^**2γ, 4**^	11.0 ± 2.8	11.5 ± 2.9^**a**^	11.8 ± 1.9	9.8 ± 1.5*^,ba^	10.3 ± 2.3	9.2 ± 1.8^**b**^
MUFA **(% fat) **^**2γ, 4**^	32.5 ± 3.8	31.7 ± 4.0^**a**^	33.9 ± 4.1	36.9 ± 3.0*^, b^	34.0 ± 3.5	37.6 ± 2.6*^, b^
MUFA **(%EN)**^3,4^	7.2 ± 2.1^**a**^	7.2 ± 1.5^**a**^	9.0 ± 2.0 ^**b**^	8.8 ± 1.3 ^**b**^	7.6 ± 2.1 ^**ab**^	8.5 ± 1.9 ^**b**^
PUFA **(% fat) **^**4**^	17.6 ± 4.9	17.7 ± 3.5^**a**^	21.2 ± 5.6	22.0 ± 2.8 ^**b**^	19.6 ± 4.0	21.0 ± 2.9 ^**b**^
PUFA **(%EN)**^**4**^	3.8 ± 1.3 ^**a**^	4.0 ± 0.8^**a**^	5.7 ± 2.0 ^**b**^	5.2 ± 0.8 ^**b**^	4.3 ± 1.1 ^**ab**^	4.7 ± 1.0 ^**b**^
n6:n3 ratio^**2,γ, 4**^	8.9 ± 3.3	6.6 ± 3.1*	8.8 ± 3.8	8.7 ± 2.5	10.7 ± 5.5	7.9 ± 2.8*
Cholesterol **(mg/d)**	214.3 ± 97.0	182.4 ± 64.1	239.0 ± 128.9	148.7 ± 43.8*	207.1 ± 104.3	152.2 ± 54.1*
Biotin **(μg/d) **^**2**^**^**^**, 4**^	8.3 ± 5.9	16.1 ± 5.7*^,a^	8.2 ± 3.9	8.5 ± 4.7 ^**b**^	9.9 ± 6.4	9.9 ± 5.1 ^**b**^
Calcium **(mg/d) **^**2**^**^**^**, 4**^	772.3 ± 243.4	1244.8 ± 108.0*^,a^	644.0 ± 122.4	1035.1 ± 72.3*^,b^	711.7 ± 202.6	449.0 ± 69.7*^,c^
Niacin **(mg/d)**	15.9 ± 6.0	13.9 ± 3.8	15.0 ± 5.3	15.4 ± 4.8	15.4 ± 3.7	15.3 ± 5.2
Pantothenic acid **(mg/d) **^**2**^**^**^**, 4**^	2.3 ± 1.1	3.41 ± 0.7*^,a^	2.2 ± 0.9	2.1 ± 0.71 ^**b**^	2.2 ± 0.9	2.4 ± 0.9 ^**b**^
Riboflavin **(mg/d) **^**2**^**^**^**,4**^	1.3 ± 0.4	1.76 ± 0.3*^,a^	1.2 ± 0.4	0.95 ± 0.3 ^**b**^	1.3 ± 0.3	1.1 ± 0.3*^, b^
Thiamin **(mg/d)**	1.2 ± 0.35	1.1 ± 0.3	1.0 ± 0.3	1.0 ± 0.5	1.2 ± 0.4	1.1 ± 0.4
Vitamin B_6 _**(mg/d)**	1.2 ± 0.5	1.3 ± 0.4*	1.2 ± 0.5	1.3 ± 0.5	1.1 ± 0.3	1.4 ± 0.6*
Vitamin B_12 _**(μg/d) **^**2**^**^**^**, 4**^	2.7 ± 1.7	3.8 ± 1.7*^,a^	3.2 ± 1.8	2.2 ± 1.0*^,b^	2.2 ± 1.1	2.3 ± 1.4 ^**b**^
Vitamin D **(μg/d) **^**2**^**^**^**, 4**^	1.8 ± 1.1	4.8 ± 2.1*^,a^	1.8 ± 0.7	0.9 ± 0.8*^,b^	1.9 ± 1.3	1.1 ± 0.8*^, b^

^1 ^Data are means ± SD; n = 61. EN, energy; SFA, saturated fatty acids; MUFA, monounsaturated fatty acids; PUFA, polyunsaturated fatty acids.

^2^Repeated measures ANOVA was performed to test the effect of time; time x treatment and treatment, confirmed with paired sample t-test was for significant differences between baseline and 12 wk. Time, *P < 0.05; time x treatment, **^**P < 0.01, ^**γ**^P < 0.05.

^3^ANCOVA to test the effect of treatment on reported baseline nutrient intake with adjustments for reported site, sex and baseline energy intake. The final models were not adjusted for baseline energy intake because the dependent variables --MUFA and PUFA as a percent of energy are already adjusted for energy intake. Pairwise comparisons with Bonferroni adjustment was used to determine differences among treatments. Values with different superscript letters are significantly different; P < 0.05.

^4^ANCOVA to test the effect of treatment on reported 12 wk nutrient intake with adjustments for site, sex, and energy intake at 12 wk. Protein, SFA, MUFA and PUFA as a percent of energy were not adjusted for energy intake. Pairwise comparisons with Bonferroni adjustment was used to determine differences among treatments. Values with different superscript letters are significantly different. P < 0.05.

### Clinical metabolites and total lipid classes at baseline and 12 wk

There were no significant time, treatment, or time x treatment effects for total cholesterol, or LDL and HDL cholesterol (data not shown) and total lipid classes (Table 3). There were significant treatment x time interactions for total circulating CE and total FFA (data not shown) due to higher plasma baseline total CE and FFA for the dairy but not calcium or placebo groups despite subject randomization into treatment groups at enrollment. However, after adjustments for age, site, sex, HOMA-IR and physical activity at baseline, total CE at baseline was no longer significantly different for the dairy group.

**Table 3:** Circulating total lipid classes of study participants at baseline and 12 wk^1^

	Lipid (nmol/mL)	
	
Lipid class	Baseline	12 wk
Cholesterol ester	3,850 ± 870	3,770 ± 830
Diacylglycerol	69.3 ± 27.5	65.6 ± 33.0
Free fatty acid	600 ± 250	540 ± 190
Lysophosphatidylcholine	170 ± 47.0	160 ± 39.0
Phosphatidylcholine	1,470 ± 350	1,500 ± 400
Phosphatidylethanolamine	185 ± 120	180 ± 100
Triacylglycerol	1,130 ± 560	1,060 ± 500

^1^Data are means ± SD; n = 60.

### Relationships between reported dietary fat intake as a percent of total fat and plasma lipidome

There was no significant time, treatment or time x treatment effects for any plasma fatty acids of CE, FFA, PC or TG. There were no significant relationships among reported intakes of MUFA and circulating lipid species at baseline. There was a significant relationship between reported intake of MUFA (% of fat) and circulating plasma CE 18:1(n-9) at 12 wk (partial r = 0.33, *P *< 0.05), however this relationship disappeared when the model was adjusted for sex. Reported intake of % SFA and % PUFA were not associated with circulating SFA or PUFA lipid species at baseline or 12 wk, respectively.

### Relationships between reported fat intake as a percent of total energy and body composition

**Figure 1:**
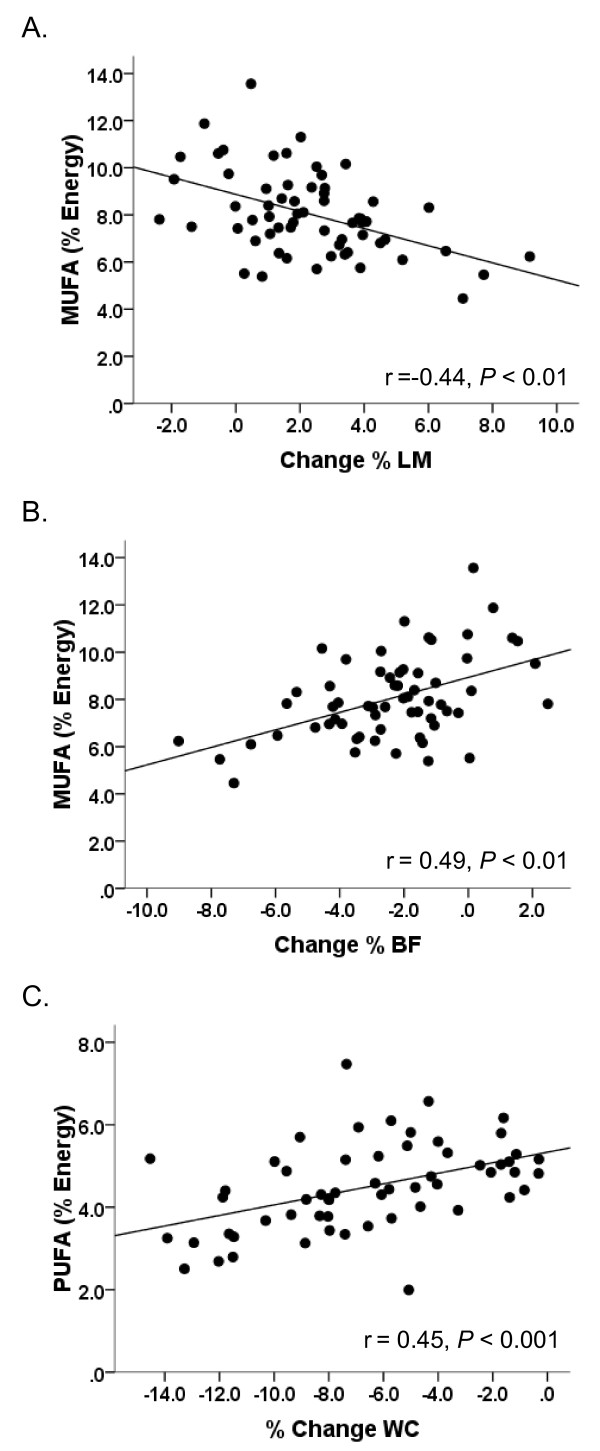
**Scatterplots of the partial correlations between reported 12 wk mean dietary fat intake (% of energy) and changes in anthropometrics**. (A) The relationship between MUFA intake and change in % LM. (B) The relationship between MUFA intake and the change in % BF. (C) The relationship between PUFA intake and the percent change in WC.

Final models for the relationships among reported fat intake and body composition are displayed in Table 4. Reported mean dietary intake of % MUFA at 12 wk was inversely associated with the change in % LM (partial r = -0.44, P < 0.01) (Figure 1A) and positively associated with the change in % BF (partial r = 0.49, P < 0.001) (Figure 1B) accounting for 44 and 49 percent of the variance in % LM and %BF, respectively. Reported 12 wk mean dietary intake of % PUFA was positively associated with the percent change in WC (partial r = 0.45 P < 0.01) (Figure 1C) accounting for 45% of the variance in percent change in WC.

**Table 4:** Final models for the relationships between dietary fat intake (% of energy) and changes in body composition at 12 wk^1^

Anthropometrics	Dietary Intake	Model R	**Adjusted R**^**2**^	Model < P
LM change (%)	MUFA	0.59	0.24	0.01
BF change (%)	MUFA	0.63	0.30	0.01
WC change (%)^2^	PUFA	0.69	0.38	0.001

^1 ^Stepwise regression (n = 61) was used to determine relationships between reported dietary fat intake and changes in body composition. Dependent variables included changes in anthropometrics and independent variables included reported intakes of SFA, MUFA and PUFA (% of energy). Final models were adjusted for site, sex, age, energy, protein (g/d), physical activity at 12 wk; and HOMA-IR at baseline. LM, lean mass; BF, body fat; WC, waist circumference.

^2 ^n = 59

## Discussion

Significant reductions in weight, WC, % BF and an increase in % LM were found among all dietary treatment groups in response to energy restriction. Dairy product consumption was found to be significantly associated with reduced WC and %BF; however, these relationships were no longer significant after adjustments for protein and energy intakes and physical activity. Circulating clinical and plasma lipidome values were not significantly associated with time or treatment. Yet, despite randomizing subjects into treatment groups the dairy group had significantly higher levels of total FFA at baseline compared with the calcium and placebo groups. This relationship was significant despite adjustments for age, site, sex, HOMA-IR, physical activity and body composition. Nonetheless, plasma total FFA at 12 wk was not significantly different among the treatment groups. Other confounding variables that could influence total FFA at baseline such as HOMA-IR, WC, body composition, age, site and physical activity were not significantly different among the treatment groups.

Based on dietary intake reported by subjects in weekly diet records, the dairy group consumed more energy, macronutrient and micronutrients such as calcium, biotin, pantothenic acid, riboflavin, and vitamin D--compared with the calcium supplement and placebo groups. Calculated dietary intakes of energy and fat composition were associated with changes in anthropometrics, but not plasma lipidome after adjustment of sex. Some caution should be acknowledged regarding the possibility of underreporting of food intake by obese subjects compounded by nutrient specific underreporting by the general population leading to unpredictable analytical consequences [36]. Furthermore, underreporting may affect the associations between dietary factors--specifically of n-6 polyunsaturated fats and fat from dairy products--and the metabolic syndrome suggesting inaccurate dietary data can introduce spurious associations [37]. In addition, Nutritionist Pro has limitations in the database for the estimation of dietary fat composition.

In addition to consuming higher micro and macronutrients, the dairy group reported significantly higher intake of SFA and lower intakes of MUFA and PUFA compared with the calcium supplement and placebo groups. Interestingly, the markers of dairy fats--myristic (14:0), --pentadecanoic (15:0) and heptadecanoic acids (17:0) [38,39] were not reflected in circulating plasma lipids of the dairy treatment group. These discrepancies are most likely due to the energy restrictive nature of this study such that subjects in the dairy treatment group were not consuming 3-4 servings of full fat dairy products. In addition, heptadecanoic acid was not included in our fatty acid output because it was one of the internal standards used in the gas chromatography analysis.

When expressed as a percent of total energy, dietary fat composition was correlated with changes in anthropometrics. Reported MUFA at 12wk was inversely and positively associated with changes in % LM and % BF, respectively. The observed associations between reported MUFA intake and body composition support *in vitro *studies that reported a positive relationship between adipose growth and differentiation in response to oleic acid incubation compared with various long-chain fatty acids [40-42] and with varying concentrations of oleic acid [42]. Unfortunately, the mechanistic relationships between dietary MUFA intake on energy partitioning and body composition *in vivo *is not well understood and this study was not designed to assess causality. Reported intakes of SFA and PUFA at 12 wk were not associated with changes in % LM, and % BF, yet PUFA intake was positively associated with the % change in WC. These data are corroborated by cross sectional studies in which PUFA intake was positively associated with body mass index and waist-hip ratio in women [43]. More specifically, intake of long chain omega 3 fatty acids were negatively associated with body fat [44] and positively associated with fat oxidation [45] while dietary omega 6 fatty acids was positively associated with body fat [44]. In this study dietary n6:n3 was not correlated with plasma lipids or changes in anthropometrics.

Our findings were unlike a previous study in which anthropometric changes were associated with dairy product consumption in a subset of the population; although adjustments for energy intake were not made [46]. In order to determine if dairy product consumption physiologically modulates body composition, several study design criteria should be addressed. In our study, the dietary treatment groups did not consume equal amounts of protein--an independent variable associated with changes in anthropometrics [47,48]. Second, there were unequal number of males and females in each dietary treatment which contributed to high inter-individual variation known to influence changes in body composition [49]. Third, attrition rates among the intervention sites were unequal which resulted in unequal number of participants for each treatment group, hence introducing variation associated with US geographical influences on habitual diet. Fourth, reported dietary intake in weekly 3-day diet records does not reliably assess dietary intake [50,51]. Fifth, the sample size of our study was fairly small unlike previous reports which identified relationships between calcium intake and anthropometrics [4]. Sixth, individuals should have been randomized into treatment groups not based on BMI or weight but by metabolic phenotype such as insulin sensitivity and or body composition to better control for metabolic responses to dietary interventions. In addition, choosing the statistical methodology for analyzing high density data needs to be addressed. One of the challenges of this study was the use of stepwise regression to identify the relationships between diet and plasma lipids and diet with changes in anthropometrics. There are limitations with stepwise regression which include: bias in overstating parameter estimation; risk of achieving a type 1 error from multiple testing; reliance on over-fitting final models [52]. Because plasma lipids are substrates and products of shared metabolic pathways, there will be collinearity. To address these limitations, modeling in this study was guided under the current understanding of lipid metabolism and diet rather than random parameter selection. In addition, specific criteria were used to ensure reliable model selection such as Cook's Distance, multi-collinearity testing and stringent stepping method criteria with a F statistic probability for entry (*P *< 0.01) and removal (*P *> 0.05) into the model. Furthermore, this study was powered to determine the change in body weight and not with respect to the relationship between changes in body composition and the circulating lipidome. A null finding in this relationship could be explained by the high inter-individual variation in plasma lipid classes and thus would require a larger sample size.

## Conclusion

In summary, energy restriction for 12 weeks was associated with changes in anthropometric measurements. Unlike previous studies that found associations between dairy product consumption and changes in anthropometrics, this study was unable to detect associations between dairy product consumption or calcium supplementation and body composition. Yet, this study supports an important role of dietary fat composition on changes in body composition during energy restriction. Future large clinical trials designed to identify phenotypic responses to diets differing in fat composition for achieving metabolic regulation and beneficial changes to body composition are warranted.

## Abbreviations

BF: body fat; CE: cholesterol ester; LM: lean mass; MUFA: monounsaturated fatty acids; ND: not determined; PC: phosphatidylcholine; PUFA: polyunsaturated fatty acids; SFA: saturated fatty acids; TAG: triacylglycerol; WC: waist circumference.

## Competing interests

The authors declare that they have no competing interests.

## Authors' contributions

JTS, analyzed and interpreted the data, wrote the manuscript; MMW, interpreted the data and critically revised the manuscript, DT, designed the trial; MBZ, designed the trial; MVL, designed the trial, critically revised the manuscript; JBG, interpreted the data, wrote the manuscript. All authors read and approved the final manuscript.
